# Potential Biomarkers and Their Applications for Rapid and Reliable Detection of Malaria

**DOI:** 10.1155/2014/852645

**Published:** 2014-04-02

**Authors:** Priyamvada Jain, Babina Chakma, Sanjukta Patra, Pranab Goswami

**Affiliations:** Department of Biotechnology, Indian Institute of Technology Guwahati, Guwahati, Assam 781039, India

## Abstract

Malaria has been responsible for the highest mortality in most malaria endemic countries. Even after decades of malaria control campaigns, it still persists as a disease of high mortality due to improper diagnosis and rapidly evolving drug resistant malarial parasites. For efficient and economical malaria management, WHO recommends that all malaria suspected patients should receive proper diagnosis before administering drugs. It is thus imperative to develop fast, economical, and accurate techniques for diagnosis of malaria. In this regard an in-depth knowledge on malaria biomarkers is important to identify an appropriate biorecognition element and utilize it prudently to develop a reliable detection technique for diagnosis of the disease. Among the various biomarkers, plasmodial lactate dehydrogenase and histidine-rich protein II (HRP II) have received increasing attention for developing rapid and reliable detection techniques for malaria. The widely used rapid detection tests (RDTs) for malaria succumb to many drawbacks which promotes exploration of more efficient economical detection techniques. This paper provides an overview on the current status of malaria biomarkers, along with their potential utilization for developing different malaria diagnostic techniques and advanced biosensors.

## 1. Introduction

The World Malaria Report 2012 released by World Health Organization (WHO) summarises the data received from 104 malaria endemic countries [1]. This report estimated around 219 million cases of malaria and a high mortality of about 660,000 people due to the disease in the year 2010. Malaria in humans is transmitted by the bite of more than thirty species of female anopheline mosquitoes. The causative agent is a protozoan parasite of genus* Plasmodium*. Five species,* P. falciparum, P. vivax, P. malariae, P. ovale, *and* P. knowlesi*, are known to affect humans. Characteristic symptoms of the disease include episodes of high fever followed by chills and rigors which are repeated every 48 hrs in* falciparum*,* ovale*, or* vivax*, every 72 hrs in* malariae* infections, and 24 hrs in* knowlesi*.* P. vivax* hypnozoites can lie dormant in the liver and may cause recurrence of the disease [2]. Indiscriminate use of oral artemisinin based monotherapies has been identified as one of the factors that lead to drug resistance, which is a serious problem in malaria management. For example, a sulfadoxine-pyrimethamine combination, which is an effective treatment against multidrug resistant (MDR) malaria, has now been rendered useless in Southeast Asia by its overuse and misuse [1, 3]. WHO recommends that all persons suspected of malaria should receive parasitological confirmation before the drugs being administered, which prevents mismanagement of nonmalarial fevers [4] and lessens overdiagnosis of malaria [5, 6].

In spite of the vast array of tests available today to diagnose malaria, we still await a test that is quantitative and specific to distinguish different* Plasmodium* species. Although rapid detection tests (RDTs) have facilitated considerably diagnosing malaria, the tests are plagued with many limitations such as variability in results, being nonquantitative, and having poor storage stability in tropical regions. In order to develop an efficient test for diagnosis of malaria, a profound understanding on various malaria-related biomarkers is prerequisite. This review outlines the diagnostic tools for malaria with special focus on the potent biomarkers reported to date and their application for developing rapid and reliable detection techniques including biosensors.

## 2. Biomarkers for Malaria

Biomarkers are cellular, biochemical, or molecular alterations measurable in biological samples which indicate any biological, pathogenic, or therapeutic responses [7]. There has been no established classification system to categorize biomarkers to date. However, Frank and Hargreaves [8] bring clarity to the biomarkers by classifying them into three types: Type 0, Type 1, and Type 2. Type 0 biomarkers are measures of the natural history of disease and correlate with clinical outcomes; Type 1 biomarkers usually determine the biological effect of a therapeutic intervention; and Type 2 biomarkers are the equivalent of “surrogacy” markers where a surrogate point has been defined as a biomarker intended to substitute for a clinical end point, with the latter being a characteristic or variable that reflects how a patient feels, functions, or survives. Biomarkers are useful for disease management as well as formulating strategies before the onset of disease in case of asymptomatic malaria. In malaria endemic areas like Africa, where transmission is high, it has been found that many* P. falciparum *infected individuals exhibit asymptomatic malaria [9]. The prevalence of these asymptomatic infections can be as high as 52%.

### 2.1. Lactate Dehydrogenase

#### 2.1.1. Metabolic Role

The red blood cell (RBC) is a nonproliferating cell with a modest requirement of around 5 *μ*mol glucose/24 hrs/10^9^ RBCs. However,* Plasmodium* is a voracious scavenger of blood glucose that increases the RBC glucose consumption up to 100-fold. In case of* P. falciparum* about 60–70% of the glucose is converted to lactic acid and excreted; however, this percentage varies amongst different* Plasmodium* species and* in vitro* culture conditions [10]. During the intraerythrocytic stages, the parasite principally relies on anaerobic respiration for ATP generation from glucose, and the NAD^+^ is regenerated by conversion of pyruvate to lactate while the mitochondria contribute minimally to the ATP pool [11]. This reaction is catalysed by lactate dehydrogenase (LDH), the final enzyme of the glycolytic pathway in* Plasmodium*. The absence of F0 a and b subunits of the mitochondrial F0-F1 ATP synthase further implies the absence of mitochondrial role in energy generation. However, since parts of the genome sequence are still not known, and the genome being A+T rich that causes difficulty in identifying enzymes, the lack of role of mitochondria in energy generation cannot be ascertained [12]. Nevertheless, due to the dependence on the glycolytic cycle for energy generation, enzymes involved in this pathway are overexpressed [13]. It has been found that* P. falciparum* LDH (PfLDH) RNA expression level gradually increases, with the peak expression being at 24 to 30 hrs in the intraerythrocytic cycle. This expression declines to zero in the schizont stage. A similar profile that slightly lags behind the RNA expression was observed for the enzyme activity as well [14]. With the help of microarray technology using* P. falciparum* transcription, it was demonstrated that all glycolytic enzymes are upregulated at the early trophozoite stage during the asexual cycle, coinciding with the time of maximal metabolic activity by the parasite [15].

#### 2.1.2. Structure and Kinetic Parameters of LDH

The parasite LDH (pLDH) is a tetramer where each monomer consists of two domain LDH folds. The larger domain comprises the Rossmann fold that binds the cofactor NADH, while the catalytic residues (His 195, Asp 168, and Arg 171) are located in the other domain. These residues are conserved across all* Plasmodium* species except* P. knowlesi*, which lacks His 195. The active site of the enzyme is located between these two domains. Each monomer of the pLDH protein carries one NADH molecule, and each of the four cofactors occupies identical positions in each monomer [16].

Protozoal LDHs display some major structural and kinetic differences compared to their mammalian counterparts that may be exploited to develop selective drugs and detection systems for malaria. PvLDH and PfLDH share 26% and 29% sequence identity with human LDH-A (hLDH-A), respectively. Structurally the parasite enzyme differs in having a five-residue insertion in its active site loop which closes down over the active site during catalysis [16] and also causes a displacement of ~1 A° of the nicotinamide moiety of the NADH cofactor [17, 18]. This insertion “DKEWN” (D-Aspartic acid, E-Glutamic acid, K-Lysin, W-Tryptophan, and N-Asparagine) was used as a common diagnostic epitope by Hurdayal et al. [19] to selectively detect pLDH from human LDH isoforms. The five amino acid insertions initially observed for* P. falciparum* [20] were later discovered in all five* Plasmodium* species and in the LDH I and II of* T. gondii* [21, 22]. However, this feature was not found in all apicomplexan parasites, for instance,* Cryptosporidium parvum* which causes cryptosporidiosis, a parasitic infection of the mammalian intestinal tract.

Kinetically, the parasite enzyme differs by not being inhibited by excess of the substrate pyruvate, a feature not shared by mammalian enzymes [23]. In the latter, substrate inhibition occurs due to the slow release of the reduced cofactor NAD^+^ from the active site due to the formation of a covalent adduct with pyruvate. Reduced substrate inhibition is attributed to a single amino acid substitution in the protozoal enzyme (Ser163Leu). The single amino acid mutation has also been exploited as a general method to reduce substrate inhibition in L-lactate dehydrogenase enzymes [24]. The substrate inhibition in plasmodial counterparts is lower by around 175- and 35-fold than that shown by the human heart and muscle isoforms, respectively [25]. The mechanism for the lower substrate inhibition is stated to be the weaker binding of pyruvate to enzyme-cofactor complex rather than slower release of NAD^+^ [16, 26]. Another kinetic difference is the ability of the pLDH to efficiently use the synthetic cofactor APAD^+^ (3-acetylpyridine adenine dinucleotide) [17, 27]. Increase in entropy on APAD^+^ binding altering the rate of active site movement or a higher oxidation potential of APAD^+^ than NAD^+^ leading to faster hydride transfer to APAD^+^ is ascribed as one of the reasons for this cofactor preference [16, 28, 29]. By using lactate and NAD^+^ as inhibitors in a forward reaction of pyruvate to lactate direction it was revealed that this enzyme exhibits ordered sequential bi-bi mechanism of binding in which the NADH cofactor binds before the pyruvate substrate is attached.

Another striking difference of pLDH from other dehydrogenases is its substrate specificity due to long substrate specificity loop. The enzyme efficiency decreases with the increase in number of methylene groups in the substrate, as is seen when pyruvate was replaced with *α*-ketobutyrate. Also the enzyme does not show an activity with phenylpyruvate [25], a behaviour that distinguishes the enzyme from the LDH of* T. gondii*. Although LDHs from* P. falciparum* and* T. gondii* have very similar structures, the latter has an additional loop insertion of two residues and several changes in its active site that renders the contrast property [29, 30]. The presence of a positively charged lysine amino acid at position 102 gives the* Toxoplasma* enzyme a mild malate dehydrogenase activity [25]; this finding, however, is not supported by the previous report [16]. A characteristic feature of this family of enzymes is that the value of *K*_*m*_ for substrates is pH dependent, as the substrate pyruvate binds only when the active site histidine is in the protonated state and lactate is in deprotonated state [31].

pLDH is an attractive target for antimalarial drug design because of its three important attributes. (1) It controls the production of plasmodial ATP, (2) it has unique amino acids at the active site compared to its counterparts from other species, and (3) the protein data bank (PDB) contains many X-ray crystallographic structures of pLDH complexed with various compounds which provide ideal targets for modelling of inhibitors (See Table S1 in Supplementary Material available online at http://dx.doi.org/10.1155/2014/852645). From the inhibition studies of various compounds including gossypol and its derivatives against the parasite enzyme pLDH [32–37] (Table S2), it has been revealed that these compounds specifically target pLDH and not human LDH. The findings underline the fact that there are structural differences between the two enzymes that may be exploited for selective detection of pLDH for malaria diagnosis.

### 2.2. Histidine-Rich Protein II

#### 2.2.1. Occurrence

For the first time in the avian malarial parasite* Plasmodium lophurae,* numerous cytoplasmic granules were isolated and studied. It was shown to have an unusual polypeptide composition whose amino acid analysis showed 73% histidine, 7.5% proline, 7% alanine, 6% glutamic acid, and 2.1% aspartic acid [38]. This unusual histidine-rich polypeptide raised interest in the structure of the protein and its function. Histidine-rich proteins (HRPs) are also observed in many organisms with different functions [39–42].

*P. falciparum* synthesizes a unique set of soluble HRPs during the asexual erythrocytic development which are denoted as HRPs I, II, and III in the order of their discovery [43]. HRP I protein also known as knob-associated protein (KAHRP-I) is found in Knob^+^ strain which means phenotypically that it expresses knob-like protrusion on the cell surface and is suggested to help in the cytoadherence of infected erythrocytes to the venular endothelium [44] and partially contributes to the high parasitemia and hypoxia associated with* P. falciparum*. HRP II, which is exclusive to* P. falciparum,* is found in both Knob^+^ and Knob^−^ strains and is reported to have many functions such as heme binding and heme detoxification by forming hemozoin [45, 46]. It has been projected as a model vaccine against malaria too [47]. HRP III also known as small histidine-alanine-rich protein (SHARP) is a smaller protein and is not found as abundant as the other two proteins. The HRP III sequence of FC27 strain, a* Plasmodium* isolate FCQ27/PNG from Papua New Guinea, was reported and shown to have polymorphisms in the gene's repeats [48]. It shares high homology with HRP II [49]. Also varying degrees of cross-reactivity between HRP II and HRP III have also been reported for HRP II MAbs: 2G12, MAb87, and 1D6 [50].

#### 2.2.2. Genetic and Structural Organization

**Figure 1:**
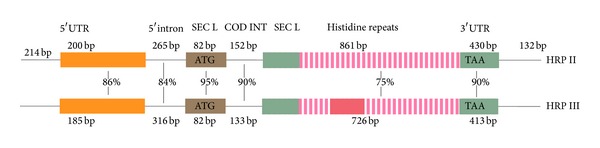
Schematic alignment of* hrp II* and* hrp III* genes including 5′ and 3′ UTRs. INT and SEC L stand for intron and secretory leader, respectively. The gene consists of a hydrophobic signal peptide (brown), an intervening intron, and an extensive region of tandem repeats (pink). The high homology (85–90%) between the tandem repeat domains and the regions flanking the repeats of* hrp II* and* hrp III* genes is shown [51].

The genomic sequence of* hrp II* contains a hydrophobic signal peptide, an intervening intron and an extensive region of tandem repeats that encodes a 35 kDa polypeptide consisting mostly of histidine, alanine, and aspartic acid. There are 18 tripeptides (Ala-His-His), 3 pentapeptides (Ala-His-His-Ala-Ala), and 33 hexapeptides (Ala-His-His-Ala-Ala-Asp). There is about 85–90% homology between the tandem repeat domains and the regions flanking the repeats of* hrp II* and* hrp III* genes which imply that both have originated in the duplication of an ancestral sequence [49]. Another study of the flanking 5′ and 3′ untranslated regions (UTR) of the* hrp II* and* hrp III* genes revealed a 5′ UTR intron which has been shown to have high conservation as the coding intron and a 3′ UTR, having a much greater homology than the histidine repeat region of the coding sequences [51].  Figure 1 demonstrates the homology between the two genes. Both* hrps II* and* III* share many similarities, but still it is unclear how these genes function and why HRP II is released in large quantities.

**Figure 2:**
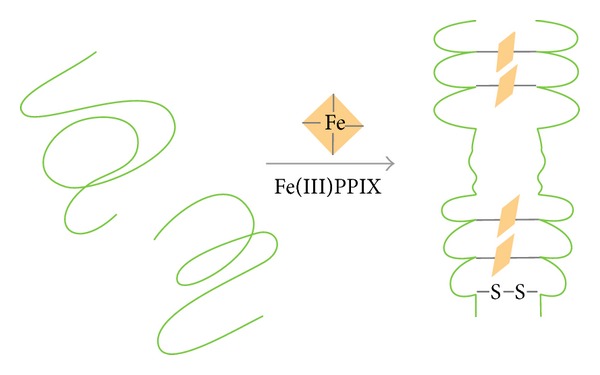
Fe^+3^ PPIX (protoporphyrin IX) binding to HRP II is able to bring about an interaction between two monomers resulting in the formation of an intermolecular disulphide bond.

HRP II transports from the parasite, through the host cell cytoplasm, to the culture supernatant* in vitro* [43] which also accounts for its presence in the serum of plasma, cerebrospinal fluid, and urine of infected patients [52, 53]. It is also present in food vacuole [54], digestive vacuole [55], and membrane surface of the infected RBCs [50]. In short, HRP II is found everywhere and released in abundance which is also a reason for its importance as an antigen biomarker of malaria. The CD spectrum of HRP II in aqueous medium was indicative of random coil and the spectrum changes dramatically from random coil to 3_10_-helix conformation as soon as heme molecules are titrated in the solution [56]. The 3_10_-helix conformation is commonly observed in proteins as short sequences of three to four residues, mostly associated with N- or C-termini of *α*-helices [57, 58]. However, the length of 3_10_-helix is found to be unusually long in case of HRP II. Along with the changes in the secondary structure of the protein, dimerization of HRP II monomers by formation of intermolecular disulfide bonds also takes place as shown in Figure 2. This process of dimerization depends on heme binding. There is no X-ray crystal structure of HRP II available till now.

#### 2.2.3. Function of HRP II

The actual function of the HRP II is not yet adequately known. However, many reports have demonstrated that its main function is heme binding that may link its role in the heme detoxification in malaria parasites. Spectroscopic studies showed that a single HRP II molecule may bind to multiple heme molecules [59]. By using biuret assay based spectroscopic method, it was determined that 15 heme molecules bind to one HRP II molecule [56]. The *K*_*d*_ values for heme binding were calculated to be 0.34 *μ*M. In another report the number of binding sites in HRP II-heme complex is estimated as 18 with *K*_*d*_ of 0.94 *μ*M [60], which is in agreement with another report [55]. Hence the estimated binding sites of heme molecules are around 15–18. Further, it was confirmed that the heme molecules in protein heme complex are in low spin, six coordinated, and bis-imidazole ligated. There are also reports on heme-artemisinin adducts. Artemisinin is an antimalarial drug that bears a peroxide function and is triggered only in parasite infected hosts. The drug binds with higher affinity to HRP II as compared to the heme moiety [61].

HRP II is also known to initiate the formation of hemozoin.* In vitro* heme polymerization assay performed at the optimum pH 4.0 with native and recombinant HRP II promoted the formation of hemozoin. Polymerization increased with time, protein concentration, and initial concentration of heme [55]. A study conducted on biomineralized dendrimeric templates showed potential of heme binding to metal moieties and initiated hemozoin formation [62]. The formation of hemozoin is, however, a complex phenomenon and needs further investigation to be understood adequately.

At an acidic pH electrostatic interaction takes place between the strongly positive charged histidine of HRP II and negative groups on actin and phosphatidylinositol 4,5-biphosphate (PIP_2_) of host cells. An important role of HRP II as a buffering protein has been suggested as it helps the parasite to stabilize the changes to the cytoskeleton induced by other released parasitic proteins [63]. Another function of HRP II is the neutralization of bacterial toxin LPS [64]. The observed LPS-neutralizing effect of HRPs has probably resulted from the electrostatic interactions between histidines and the negatively charged phosphate groups of LPS.

### 2.3. Hemozoin

Hemozoin is an insoluble microcrystalline product formed from the digestion of blood by* Plasmodium *spp. and few other species of blood-feeding parasites [65–67]. Originally, Giovanni Maria Lancisi (1717) had observed that internal organs of malaria victims are decolorized; the biochemical reason of it was, however, not known. Later on the cause was attributed to hemozoin. Hemozoin plays a role as visible marker in identifying malarial parasites and hence, it is popularly termed as malaria pigment. The parasites infect the RBCs and digest hemoglobin resulting in release of amino acids and toxic-free heme (ferriprotoporphyrin IX) which is polymerized to hemozoin. The crystal structure of hemozoin consists of an unusual polymer of hemes linked between the central ferric ion of one heme and a carboxylate side-group oxygen of another heme [68]. Later on its crystal structure was confirmed to be similar to synthetic *β*-hematin using X-ray diffraction powder pattern [69]. Though there has been extensive study on the structure and characterization of hemozoin crystals, the process of its nucleation is not yet clearly known. There are reports about initiation of hemozoin formation occuring autocatalytically, while other reports suggest that it requires external aids [70]. Most popular belief is that HRP II helps in the initiation of hemozoin formation as it binds firmly to heme molecule in aqueous environment. However, there are other mechanisms that support the view that it is promoted by polar membrane lipids [71] and neutral lipid bodies [72–74]. Since it is a survival tactic of the parasite from the heme toxicity, it has been targeted for malaria drug study for many years [75].

### 2.4. Other Biomarkers

#### 2.4.1. Aldolase

Aldolase is a key enzyme in the glycolytic pathway of the parasite. It catalyses the cleavage of fructose-1,6-bisphosphate into glyceraldehyde-3-phosphate and dihydroxyacetone phosphate [76]. The genomic sequence of aldolase consists of two exons interrupted by one intron. The first one encodes only one amino acid, the initiation methionine, while the second one encodes the residual 368 amino acids of the protein. The enzyme is homotetrameric protein with each subunit of approximately 40 kDa [77]. Crystal structure of plasmodium aldolase has been studied at a resolution of 3 A° to determine possible structure based inhibitors [78]. The aldolase is localized in the cytoplasm of the parasite as an active and soluble form and is also found to be associated with the membrane fraction as an insoluble form. The enzyme has high degree of sequence diversity from the host and thus has the potential as a drug target. There have been studies on inhibition of recombinant* P. falciparum* aldolase by several candidates like rabbit antibodies, a 19-residue synthetic peptide, and phosphorothioate antisense oligodeoxynucleotides [77, 79]. In higher vertebrates there are three tissue-specific aldolase isoenzymes whereas only one aldolase enzyme has been identified in blood sucking parasites, for example,* Trypanosoma brucei* [80],* Giardia lamblia* [81], and* P. falciparum* [82], suggesting that multiple isoenzymes are not necessary for the completion of protozoan parasite life cycles. In an experiment examining stage-specific expression of aldolase isoenzymes in* P. berghei*, two genes of aldolase (aldo-1 and aldo-2) were found where aldo-1 sequence was found to be identical to* P. falciparum* aldolase, whereas aldo-2 had 13% sequence diversity [83]. In another study the above statement was contradicted and it was reported that only single aldolase gene occurs in the following malaria species:* P*.* berghei*,* P. chabaudi*,* P. vinckei*, and* P. yoelii* and from the human malaria parasite* P. vivax* [84]. Many reports have shown poor sensitivity of aldolase RDTs which encouraged studying more its genetic diversity. Lee et al. [85] studied the diversity in* P. falciparum* and* P. vivax* aldolase and showed that aldolase is highly conserved, indicating that antigenic diversity is not a cause of variable RDT sensitivity.

#### 2.4.2. Glutamate Dehydrogenase

Glutamate dehydrogenases (GDHs) are ubiquitous enzymes that occupy an important branch point between carbon and nitrogen metabolism. They are generally involved in ammonium assimilation (NADP-dependent GDHs) or glutamate catabolism (NAD-dependent GDHs).* P. falciparum* express three GDH isozymes. PfGDH1 is a NADP-dependent glutamate dehydrogenase which is a homohexamer with a subunit of *M*_*r*_ 49,500 [86]. It has been postulated to play a role in the parasite's redox metabolism [13]. The crystal structures of PfGDH 1 and 2 have been solved to a resolution of 2.7 A° [87] and 3.1 A° [88], respectively. GDHs possess a unique N terminal residue extension not found in the mature human enzyme and are present throughout the intraerythrocytic cycle of the parasite. Furthermore GDHs are absent in the host RBC making them a potent biomarker [86]. PfGDH1 was used to detect the presence of* P. falciparum* using western blotting [89]. Monoclonal antibodies in combination with colloidal gold were used in an immunochromatographic assay for diagnosis of* P. falciparum*. This assay showed a sensitivity and specificity of 86.6% and 96.4%, respectively [90].

#### 2.4.3. Serological Biomarkers for Cerebral Malaria

Cerebral malaria (CM) is a life-threatening complication of malaria and is defined as an unarousable coma with a* P. falciparum* infection in the absence of other causes of encephalopathy. If left untreated, it is fatal within 24–72 hrs [91]. The advantage of an early immunologically relevant serological biomarker for CM would be stratifying febrile patients into groups: those who are likely to develop CM and those who are likely to develop severe malaria (SM) or mild malaria (MM). Although no clear biomarkers have yet been identified for CM, studies show a conspicuous relationship between chemokine interferon inducible protein (CXCL10 and CXCL4) and severity of CM [12, 92]. It was observed that patients with CM have significantly elevated levels of CXCL-10 and CXCL-4.

Several other serological biomarkers for CM have also been identified. For example, elevated levels of soluble tumor necrosis factor receptor (sTNF-R) and soluble Fas ligand (sFas), a 26 kDa glycoprotein generated from its membrane bound form by a metalloproteinase-like protease, may indicate CM neuropathy [92]. Cerebrospinal fluid (CSF) and serum levels of twelve cytokines and chemokines were monitored and it was found that elevated levels of interleukin 8 (IL-8), interleukin 1 receptor antagonist (IL-1ra), and TNF*α* were present in children infected with CM as compared to healthy controls [93]. The levels of endothelial regulators like angiopoietin I (ANG I) and ANG II are also affected during CM. It was found that ANG II and ratio of ANG II: ANG I could be used to accurately distinguish between CM and MM patients. This study was done with serum [94] as well as whole blood [95] angiopoietin levels. Potency of a neuroprotective factor, erythropoietin (EPO), was assessed to predict the risk of developing neurological sequelae after recovery to CM [96]. EPO has also been used for adjunctive therapy to prevent neuronal damage [12] in addition to its use as a prognostic biomarker. Serum levels of soluble adhesion molecules, like intracellular adhesion molecule I (ICAM-I), vascular cell adhesion molecule I (VCAM-I), and so forth, were studied in Gambian children and were found to be elevated in children with SM compared to MM [97]. Kassa et al. [98] have exploited the amphipathic nature of hemozoin to capture serum proteins that differ between malaria-infected individuals and healthy individuals. LC-MS/MS analysis of the captured proteins showed that serum amyloid A (SAA), apolipoprotein E (ApoE), and lipopolysaccharide (LPS) binding protein may have potential to act as prognostic biomarkers. Complement system components like C1q, C3, C4, and C5a are affected in malaria patients. Depressed levels of C3 were found in severe and uncomplicated malaria as compared to healthy controls [12, 99].

Microparticles (MPs), also known as microvesicles, are fragments of plasma membrane shed by various cell types under physiological stress conditions and have also been linked with pathophysiology [100]. MPs have been reported in the serum of patients suffering from malaria and diabetes [101, 102], systemic lupus erythematosus [103], and acute coronary syndromes [104] and in conditions of severe trauma [105]. There is a dramatic difference in the plasma levels of MP of endothelial origin among Malawian children suffering from CM, severe malarial anaemia, and uncomplicated malaria caused by* P. falciparum* [106]. Mfonkeu et al. [107] demonstrated by using fluorescence activated cell sorting (FACS) that MP of platelet origin may be a relevant marker in the routine followup since the levels of MP dramatically increased during CM and decreased when the patient was cured.

The serological biomarkers, however, are difficult to use reliably in diagnosing malaria. For instance, CXCR3 and its ligands have been implicated in case of several neurological diseases like West Nile virus [108, 109],* Toxoplasma gondii* [12], and HIV infections [110]. Serological biomarkers may be used to indicate CM only after the malaria diagnosis is confirmed using an antigenic biomarker. Potential diagnostic antigenic biomarkers have been summarised in Table 1 for better understanding.

## 3. Detection of Malaria

### 3.1. Microscopic and Conventional Analytical Techniques

Microscopy for malaria diagnosis has been used since 1904 when Gustav Giemsa introduced a mixture of methylene blue and eosin to stain the parasite [113]. This method is, however, time consuming and labour intensive and requires expert skills. To enhance the sensitivity of microscopic detection, fluorescent microscopy using fluorophores such as acridine orange and benzothiocarboxypurine has frequently been used in addition to quantitative buffy coat technique. Although microscopy was considered as “gold standard” for malaria diagnosis, recent reports suggest otherwise. Bayesian latent class models were used to estimate sensitivities and specificities of various diagnostic tests and revealed that microscopy is a poor reference test [114].

Apart from microscopy, nucleic acid techniques such as PCR and nested PCR [120, 121] and LAMP have also been used for malaria detection. LAMP can be conducted under isothermal conditions and does not need expensive thermocyclers [120]. However, this method may pose danger of cross-contamination during addition of dye for visualization of results. Also this method is not suitable for target DNA greater than 500 bp, as this causes a hindrance to strand displacement [122]. Most of these conventional methods have been summarised in Table 2.

Flow cytometry (FCM) and automated blood cell counting techniques can be used to detect hemozoin or* Plasmodium* dsDNA in infected erythrocytes; however, the use of* Plasmodium* dsDNA as a marker in flow cytometry can result in false positives because pathological conditions are characterized by an efflux of normoblasts and erythroblasts in blood [123]. LDMS is used for detection of heme in hemozoin. Hemozoin strongly absorbs UV light resulting in vaporization of individual heme molecules. LDMS only detects small molecules (<1.5 kDa) and favours phospholipids and porphyrins; hence, it is ideally suited for heme detection; however, the detection is only semiquantitative and cannot differentiate between species [124].

A unified sandwich ELISA targeting PfLDH and HRP II was reported that allowed concurrent measurement of the biomolecules [125]. ELISA is an efficient method to detect malaria in a short time frame. Despite the advantages such as high sensitivity, the application of ELISA remains restricted to research settings and blood bank screening where large number of samples have to be screened every day, since there are no commercial species-specific ELISA tests kits for all species [126].

### 3.2. Rapid Detection Approaches

#### 3.2.1. Rapid Diagnostic Tests (RDTs)

RDTs are quick and portable immunochromatographic dipsticks whose sensitivity generally reaches >95% at* P. falciparum* density of 1000–2000 parasites/*μ*L [127]. They rely on capture of parasite antigen from peripheral blood using monoclonal antibodies conjugated to either a liposome containing selenium dye or gold particles. A second monoclonal antibody applied to a strip of nitrocellulose acts as the immobile phase. The antigen-antibody complex in the mobile phase migrates along the strip and is captured by the monoclonal antibody of the immobile phase, thus producing a visible coloured line [128].

Dipsticks can be divided into two classes based on their target antigenic biomarkers, namely, HRP II (only for* P. falciparum*) and pLDH. Most commercial RDTs that detect* P. falciparum* target HRP II and few others target pLDH. To enhance the efficiency of malaria detection, RDTs targeting many biomarkers at the same time are prepared. The combined immunochromographic-malaria dipstick (ICT) targets HRP II and aldolase for the diagnosis of* P. falciparum* and* P. vivax* [111, 112]. The authors suggest using aldolase based RDT for monitoring response to therapy as it is sensitive only at higher parasitemia in sample. CareStart (Access Bio, Princeton, NJ, USA) Malaria HRP II/pLDH Combo Test performed well for detection of* P. falciparum* (based on HRP II) and pan LDH for non-*falciparum* infections, but sensitivities for* P. ovale* and* P. malariae* were poor [129].

Dipstick operation is varied and may include dipping a nitrocellulose test strip into a blood specimen, placing a blood drop directly on the test strip, or onto a sample pad impregnated with labelled monoclonal antibody (mAb). Results are usually obtained in 5 to 15 minutes [130]. In many ways ICTs appear ideal: they are rapid (<30 mins) and can be easily used by healthcare workers or semiskilled volunteers.

Variations in RDT performance have been seen with season, year, age of patient, and presence or absence of fever during consultation [131]. The most common cause of poor performance of RDTs on the tropics is exposure to high temperature/humidity [132]. The loss in activity is attributed to damage to monoclonal antibodies or the nitrocellulose membrane. This was further confirmed by Chiodini et al. [133] who tested five RDTs from the same lot but stored at different temperatures of 35°C, 45°C, and 60°C. Although dipsticks are widely used, they have certain limitations such as poor species and stage differentiation and quantification, false positive results due to persistent HRP II antigenemia [134, 135] and rheumatoid factors in some patients [136, 137], and false negative results due to excess antigen in case of high parasitemia [138] or HRP II gene deletion [139–141]. The sensitivity of Parascreen (Zephyr Biomedical Systems) was found low for* P. falciparum* infections. This RDT was not acceptable for malaria diagnosis under the field conditions in the Peruvian Amazon due to HRP II and HRP III gene deletions in the malaria parasite genome [142–144]. Since HRP II persists after parasite clearance, the presence or absence of pLDH appears to be a more reliable diagnostic target, as it is produced by live parasites [118, 139, 145]. HRP II antigenemia is suspected to be a result of gametocytemia [146].

Genetic polymorphism may lead to false negative results as was observed when a countrywide assessment of polymorphism was carried out for HRP II, pLDH, and aldolase genes in Madagascar. Higher levels of polymorphism were observed for HRP II and HRP III genes predicting that about 9% of Malagasy isolates could not be detected at parasite densities <250 parasites/*μ*L [147]. This genetic variation was seen both within and among different countries. Logistic regression analysis predicted that due to this variation only 84% of the* P. falciparum* infections in the Asia-Pacific region are likely to be detected at densities <250 parasites/*μ*L. HRP III is also suspected to play a role in the performance of HRP II based tests [148]. However, a study on the assessment of plasma concentration, and hence the disease severity, has indicated that sequence polymorphism is not a significant cause of variation in HRP II concentration in plasma samples from African children [149]. In African children it is a major challenge to distinguish severe falciparum malaria from other severe febrile illnesses with* P. falciparum* infection. Plasma HRP II has prognostic significance and provides a tool to assess the risk of “true” severe malaria as compared to other severe illnesses in parasitemic African children [150]. This justifies the development of plasma HRP II concentration as a method for assessing severe falciparum malaria in African children. Several reviews outline various RDTs [151] and their evaluation reports [130]. In spite of over 100 such reports their comparative assessment is, however, difficult due to difference in population sizes, their clinical and epidemiological characteristics, different trial guidelines, and so forth [152]. A comparison of sensitivity and specificity of few of the field evaluations done during the last decade is shown in Table 3. It can be observed from the table that the sensitivity and selectivity of RDTs significantly vary over different studies and thus there is a need for more sensitive, selective, and reliable methods for rapid detection of malaria. One such effort was made by Peng et al. [153] by developing the Wondfo rapid diagnostic test which is a nanogold based immunochromatography assay that uses monoclonal antibodies. It is a more rapid and sensitive parasite detection method and results showed very good concordance with microscopy examination with a sensitivity and specificity of 95.49% and 99.53%, respectively.

#### 3.2.2. Advanced Techniques: Biosensors

A biosensor is a self-contained integrated device that is capable of providing specific quantitative or semiquantitative analytical information using a biological recognition element that is retained in direct spatial contact with a transduction element [165]. The biorecognition elements commonly reported for developing biosensors are enzyme, antibody, aptamer, DNA, and cells. The transduction principles widely used to develop biosensors are electrochemistry, piezoelectricity, and optical spectroscopy. Biosensor research holds promise for developing stable portable devices for rapid, sensitive, selective, reproducible, and economical detection of many analytes of clinical importance. It has the advantage of being used by semiskilled operators in point of care testing and by patients themselves. However, research on biosensors for detecting malaria is in nascent stage as evident from the limited number of the publications in this area. The biorecognition systems reported so far for developing malaria biosensors are mostly confined to aptamer and antibody. Aptamers are single-stranded nucleic acids (DNA, RNA, and modified RNA or DNA) that can uniquely bind to a ligand with high affinity and specificity. Unique aptamer candidates are generally picked up from 10^12^ to 10^15^ combinatorial oligonucleotide libraries over multiple rounds of* in vitro* selection, the process commonly known as SELEX [166]. Aptamers offer several advantages over the conventional antibodies; for instance, SELEX allows greater control over aptamer binding conditions, robustness of phosphodiester backbone renders them more stability as compared to protein based antibody, and they can be easily amplified using PCR and are highly specific [167]. It is due to these advantages that aptamer is gradually replacing antibody as the biorecognition element. The biomarkers against which aptamer or antibody has been raised to develop malaria biosensors till now are limited to pLDH and HRP II. The prominent malaria biosensors reported so far are briefly described below.

**Figure 3:**
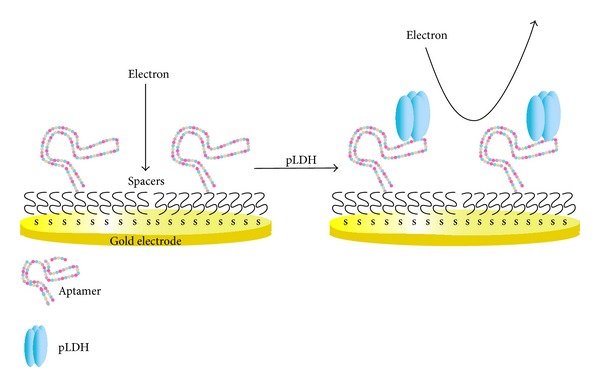
Detection of pLDH by electrochemical impedance spectroscopy. The aptamer is shown as a chain of different coloured circles, representing the four bases A, T, G, and C. Capture of pLDH by aptamer results in a decrease in electron transfer to electrode. The pLDH aptasensor can distinguish between malaria positive blood samples of two major species (*P. vivax* and* P. falciparum*) and has a detection limit of 1 pM. [115].

*(1) Electrochemical Biosensors.* Lee et al. [115] had developed an aptasensor targeting pLDH, the schematic of which is shown in Figure 3. Electrochemical impedance spectroscopy was the detection platform because of its sensitivity [168] and label-free attributes [169]. The detection limit reported with the sensor was 108.5 fM and 120.1 fM for PvLDH and PfLDH, respectively [115].

**Figure 4:**
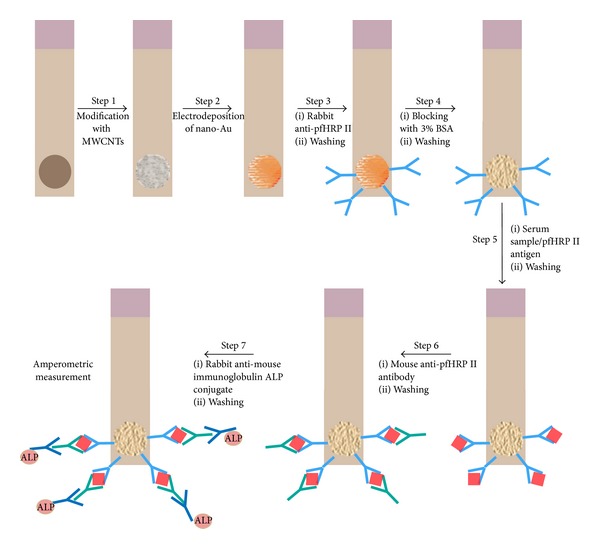
Fabrication of a sandwich immunoassay using screen-printed electrodes (SPEs) modified with gold nanoparticles and carbon nanotubes. This approach used ALP-conjugated antibodies which produce 1-naphthol as the hydrolyzed product of 1-naphthylphosphate (the enzymatic substrate) for the amperometric detection of HRP II [116]. BSA: bovine serum albumin; ALP: alkaline phosphatase.

Different electrochemical immunosensors have also been developed using antibody raised against HRP II. The first disposable amperometric HRP II based immunosensor was developed for the detection of* P. falciparum* malaria. To construct the immunosensor, disposable screen-printed electrodes (SPEs) were modified with multiwall carbon nanotubes (MWCNTs) and Au nanoparticles as shown in Figure 4. Nano-Au/MWCNT/SPEs yielded the highest-level immunosensing with a detection limit of 8.0 ng/mL. The amperometric immunosensor was compared to a commercial RDT Paracheck Pf kit (Orchid Biomedical Systems) keeping microscopy as gold standard. The Paracheck Pf kit exhibited a sensitivity of 79% and a specificity of 81% whereas the developed amperometric immunosensor showed a sensitivity of 96% and a specificity of 94% [116].

**Figure 5:**
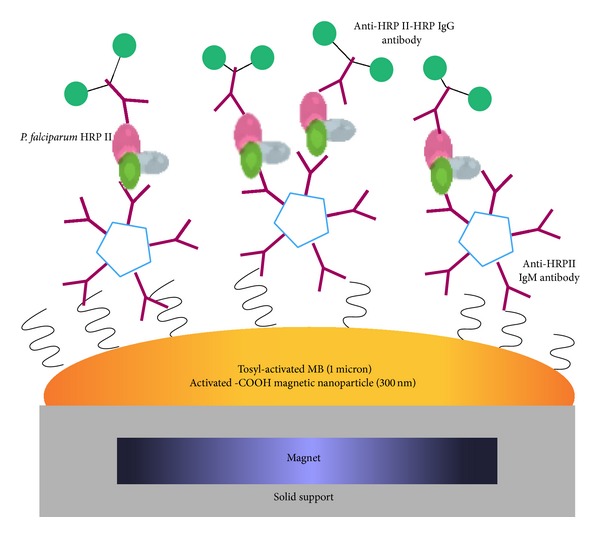
Detection of HRP II based on a magnetic sandwich immunoassay performed on magnetic beads or nanoparticles modified with monoclonal anti-HRP II IgM antibody. Detection is done using a monoclonal IgG antibody labelled with horse radish peroxidase to obtain an optical or electrochemical signal [117].

An immunosensor based on magnetic micro- and nanoparticles were also developed for targeting HRP II [117], the configuration of which is shown in Figure 5. A sandwich assay was performed on both kinds of particles by using a secondary monoclonal antibody labeled with the horseradish peroxidase (HRP) enzyme. Both optical and electrochemical detection methods were studied and compared. The electrochemical magneto-immunosensor coupled with magnetic nanoparticles had shown better analytical performance in terms of detection limit (0.36 ng mL^−1^).

**Figure 6:**
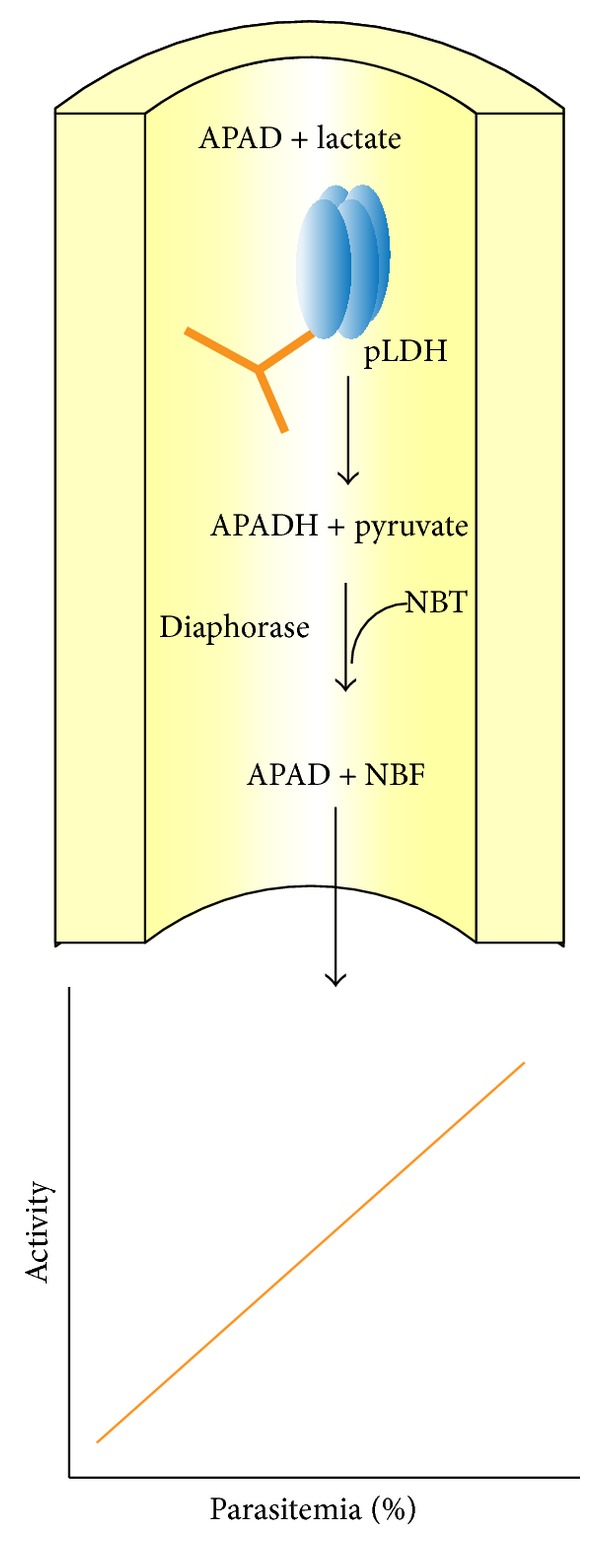
The immunocapture* Plasmodium* lactate dehydrogenase (ICpLDH) assay. A schematic of the reaction is shown in which the pLDH is immobilized using a monoclonal antibody. The enzyme activity can be measured using a coupled enzyme assay that generates APADH. The latter reduces nitro blue tetrazolium, a chromogenic substrate, using an enzyme diaphorase. Activity is quantified spectrophotometrically at 650 nm and plotted as a function of percentage parasitemia [118].

*(2) Optical Biosensors.* An immunocapture spectrophotometric diagnostic assay based on PfLDH, which showed a detection limit of 50 parasites/*μ*L and sensitivity of 88%, has been developed by Piper et al. [118]. A schematic of this assay is shown in Figure 6. This assay could be further recreated to develop an advanced biosensor with enhanced selectivity. Since it incorporates the functional activity of PfLDH in a secondary enzymatic reaction for generating signal, this assay considerably reduces the number of false positives, which may occur on nonspecific binding of serum proteins to antibodies.

Besides PfLDH and HRP II, hemozoin has also been used as a biomarker for detecting malaria microscopically. In fact, the characteristic morphology of the crystals can also be considered to distinguish different species of parasites [170]. Hemozoin crystals are paramagnetic in nature and hence show cotton-mouton effect in the presence of an external magnetic field which corresponds linearly to the hemozoin concentration [171]. Based on this concept an* in vivo* diagnostic method using a magnetooptic fingertip probe was developed and the method was confirmed by a small preliminary clinical trial [171, 172]. Following this method the hemozoin concentrations of less than 0.02 *μ*g/mL could be detected.

**Figure 7:**
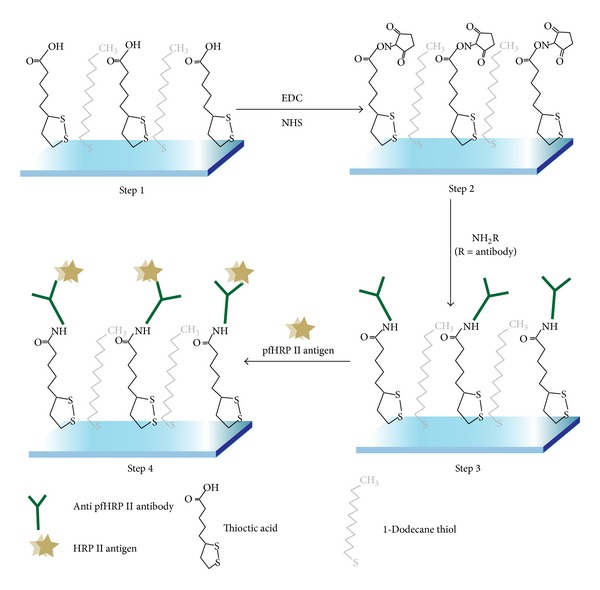
Schematic diagram for preparation of piezoelectric immunosensor for HRP II. The mixed self-assembled monolayers (SAMs) of thioctic acid and 1-dodecanethiol were formed on gold surface of quartz crystal. The rabbit anti-PfHRP II antibodies were coupled on mixed SAM modified gold surface of quartz crystal via NHS/EDC activation method. The amount of HRP II molecules bound on the sensitive area of the electrodes is quantitatively measured as a decrease in resonant frequency [119].

*(3) Piezoelectric Biosensors. *A piezoelectric immunosensor was developed using mixed self-assembled monolayers (SAMs) of thioctic acid and 1-dodecanethiol, the schematic configuration of which is shown in Figure 7. The characteristics of the immunosensor applied to detect HRP II are linear range of 15–60 ng/mL, minimum detection limit of 12 ng/mL, and storage stability (*t*_1/2_) of 14 days. The sensor was tested on clinical human serum samples and the results were compared with the commercially available ICT kit (NOW) which showed good agreement [119].

The malarial biosensor research, as evident from the above references, is more inclined towards electrochemical based biosensors and the reason may be attributed to some advantages of this biosensing platform; for example, label-free, portable, and small sample volume is needed for the analysis. From the sensitivity point of view electrochemical biosensors targeting HRP II and pLDH showed sensitivity even in the femtomolar range as compared to the other biosensors (Table 2). Comparing the two biorecognition elements exploited for developing malaria biosensor, namely, antibody and aptamer, the latter showed better performance in terms of sensitivity while the specificity of the aptamer developed by Lee et al. [115] is yet to reach the desired level as it could not discriminate between PfLDH and PvLDH.

## 4. Conclusion and Future Perspective

In the last two decades, biomarker research on malarial pathogenicity has taken a leap as witnessed from the discovery of many interesting molecules and functional entities which are significantly distinct from the host counterpart and other host pathogens. Among the biomarkers, pLDH, HRP II, aldolase, and hemozoin formation have received increasing attention for their possible utilizations as drug target and for developing malaria detection techniques. The increase in drug resistance of malaria parasites at alarming rates, caused primarily by the indiscriminate use and overprescription of drugs, has made the current treatment protocol inefficient. Therefore, a rapid, selective, sensitive, and less expensive detection technique is a need of the hour for quick parasitological confirmation in the patients before the administration of drugs. Though RDTs are a milestone in malaria detection due to their rapid detection capability and portable nature, these cannot be exclusively relied upon for reproducible and unambiguous results. In this regard, biosensors have emerged as a reliable diagnostic technique for malaria due to many inherent advantages set by their tenet including reusability, quantitative nature, enhanced signals at low sample volume, and better storage and operational stability. As revealed from the studies, every biomarker has some strengths and limitations. In order to develop an efficient biosensor, the strength of a biomarker needs to be exploited for which a profound understanding on the physiology and metabolism is essential, on the basis of which the biomarker is being instituted. Biosensor development and its confluence with biomarker discovery are therefore important to reach the desired goal in diagnosis and treatment of malaria.

## Supplementary Material

Table S1 : List of PDB entries for Plasmodium lactate dehydrogenase.
Table S2: List of inhibitors developed against lactate dehydrogenase from P.falciparum.

## Figures and Tables

**Table 1:** A brief summary of diagnostic antigenic markers of malaria.

Name of biomarker	Chemical nature	Localization	Salient features	References
pLDH	Homotetrameric protein with each monomer of 34 kDa	Inside infected RBCs	Presence of five amino acid residue insertions (DKEWN) in active site loop. Ability to actively utilize synthetic cofactor APAD^+^. Reduced pyruvate substrate inhibition.	[16, 17, 25]

HRP II	A 35 kDa protein	Secreted in serum of infected patient	Unique tandem repeats (Ala-His-His-Ala-Ala-Asp). 3_10_-Helix conformation when bound to heme. Secreted in abundance in serum, CSF, and urine of infected patients.	[49, 56]

Hemozoin	*β*-Hematin	Inside digestive vacuole of parasite	Consists of Fe(III)PPIX centrosymmetric dimmers linked by hydrogen bonds. Accumulates in the digestive vacuole of parasite and appears as cluster as observed under electron microscope.	[69, 75]

Aldolase	Homotetrameric protein with each subunit of 40 kDa	Inside infected RBCs	High sequence diversity from host and has potential as a drug target. Used for following response to therapy as it is detected only at high parasitemia.	[77, 111, 112]

pGDH	Homohexameric protein, with each monomer being 49.5 kDa	Inside infected RBCs	Plays a role in parasite's redox metabolism. Not found in host RBC making it a potent biomarker. Appears throughout the erythrocytic cycle.	[13, 86]

**Table 2:** Comparison of conventional detection techniques with advanced biosensors for malaria detection.

Types of test	Principle of the method	Instrument used	Sensitivity and specificity	Detection limit (parasites/*μ*L)	Response time (min)	Instrument cost
Peripheral blood smears (PBS)	Morphological changes in the stages of parasite by thick and thin blood smears	Optical microscope	Depends on the instrument quality and the skill of the handler	5–10	30–60	Low
Quantitative buffy coat (QBC)	Blood staining by acridine orange	Epifluorescent microscope	Higher than PBS test	<15	>5	Moderate
RDTs	Detection based on antigen-antibody interactions and enzyme assay	Disposable dipsticks	Moderate at higher parasitemia (>100 parasite/*μ*L)	50–100	10–15	low
PCR	Specific amplification of malaria DNA	Thermocycler	High	≥1	45–360 depending on the methods	High
Serological tests	Detection of malaria antigen or antiparasite antibodies in blood	Elisa, WB	Relatively high	30–60	Not mentioned	Moderate
LAMP	Detection of turbidity after amplifying DNA sequences	Turbidity meter	High	<60	>5	Moderate
Microarrays	Hybridization of DNA and quantification by fluorescent based detection	DNA chip	High	<60	Not mentioned	High
Flow cytometry	Detection of hemozoin	Flow cytometer	Variable sensitivity, high specificity	<1/sample	Poor correlation with parasitemia	High
Automated blood cell counters	Detection of hemozoin in activated monocyte	Hematology analyzers	Variable sensitivity and specificity	<1/sample	5–20	High
Mass spectrometry	Identification of heme	Laser desorption mass spectrometry	Undetermined	<1/sample	100 for whole blood	High
Amperometric immunosensor	Based on detecting target (HRP II) with the help of antibodies and modified electrodes that generate electricity on interaction	Cyclic voltammetry	Sensitivity is >95% and specificity >90%	8 ng HRP II/mL	Not mentioned d	Moderate
Piezoelectric immunosensor	Based on antibody interactions with target HRP II on the quartz crystal	Cyclic voltammetry	Less sensitive than amperometric immunosensor	12 ng HRP II/mL	Not mentioned	Moderate
Magneto- immunosensor	Based on antibodies targeted to HRP II which was successfully performed in a sandwich assay on magnetic micro- and nanoparticles	Amperometric controller and Microplate reader for optical measurements	High sensitivity	0.36 ng HRP II/mL	Not mentioned	Moderate
Aptasensor	Based on detecting LDH	Electrochemical impedance spectroscopy	High sensitivity	108.5 fM for PvLDH and 120.1 fM for PfLDH	Not mentioned	Moderate

**Table 3:** Comparison of evaluation reports of various RDTs done in the recent years.

Dipstick	Standard	Population	Sensitivity (%)	Specificity (%)	Reference
CareStart (Access Bio, Princeton, NJ, USA)	GTTS*	Southwestern Uganda	95.6	91.5	[154]
Vistapan (Mitra, New Delhi, India)			91.9	89.6	
Parabank ( Orchid/Zephyr, Goa, India)			84.7	94.3	
Paracheck pf (Orchid/Zephyr, Goa, India)			94	87.3	
Optimal-IT (DiaMed, Cressier, Switzerland)	GTS**	Gabon Children under 11 years	94	97	[155]
Acon (Acon Labs, San Diego, CA, USA)			94	90	
PALUTOP+4 (ALL.DIAG, Strasbourg, France)	GTTS* and PCR	Madagascar	95.4	97.1	[156]
Optimal-IT (DiaMed, Cressier, Switzerland)			75.8	99.0	
ParaHIT *f* test (Span Diagnostic Ltd., Surat, India)	GTTS* and PCR	Tanzania	69.2	100	[157]
Malaria Pf	GTTS*	Uganda	98	72	[135]
Paracheck Pf (Orchid Biomedical Systems, Goa, India)	GTTS*	Kenya	91.7	96.7	[158]
Malar-Check_*Pf *test	GTS**	Brazil	97.4%	88.5%	[159]
Makromed Dipstick Assay	PCR	Canada	97.0%	96.0%	[160]
ParaSight-F (Becton Dickinson, USA)	Thin blood smears and Quantitative Buffy Coat malaria test	France	94%	99%	[161]
ParaSight-F (Becton Dickinson, USA)	Microscopy	Iquitos, Peru, and Maesod, Thailand	95%	86%	[162]
Paracheck Pf (Orchid Biomedical Systems)	Microscopy	India	93%	84%	[163]
ParaHIT-f (Span Diagnostics)	GTS**	Tanzania	90.7%	73.5%	[164]
ParaHIT-f (Span Diagnostics)	Microscopy	India	87.5%	97%	[163]

**Giemsa thick smear.

*Giemsa thick and thin smear.

## Abbreviations

ACT: Artemisinin based combination therapies
ApoE: Apolipoprotein E
WHO: World Health Organization
RDT: Rapid detection tests
MDR: Multidrug resistant
FACS: Fluorescence activated cell sorting
RBC: Red blood cell
ATP: Adenosine triphosphate
NAD+: Nicotinamide adenine dinucleotide (oxidized)
LDH: Lactate dehydrogenase
PfLDH: Plasmodium falciparum lactate dehydrogenase
PvLDH: Plasmodium vivax lactate dehydrogenase
pLDH: Parasite lactate dehydrogenase
RNA: Ribonucleic acid
NADH: Nicotinamide adenine dinucleotide (reduced)
APAD: 3-Acetylpyridine adenine dinucleotide
PDB: Protein data bank
HRP: Histidine-rich proteins
mcfp-4: Mytilus californianus foot protein 4
HRG: Histidine-rich glycoprotein
MA: Monoclonal antibodies
UTR: Untranslated regions
CD: Circular dichroism
pGDH: Parasite glutamate dehydrogenase
PfGDH: Plasmodium falciparum glutamate dehydrogenase
CM: Cerebral malaria
SM: Severe malaria
MM: Mild malaria
IL: Interleukin
TNF: Tissue necrosis factor
ANG: Angiopoietin
EPO: Erythropoietin
ICAM: Intracellular adhesion molecule
LC-MS/MS: Liquid chromatography-mass spectrometry/mass spectrometry
SAA: Serum amyloid A
LPS: Lipopolysaccharide
PM: Placental malaria
MP: Microparticles
CXCR3: Chemokine (C-X-C motif) receptor 3
PCR: Polymerase chain reaction
LAMP: Loop-mediated isothermal amplification
IFA: Immunofluorescence antibody
FCM: Flow cytometry
LDMS: Laser desorption mass spectrometry
TDR: Tropical disease research
FIND: Foundation for Innovative New Diagnostics
CDC: Center for Disease Control and Prevention
ICT: Immunochromographic-malaria dipstick
ELISA: Enzyme-linked immunosorbent assay
SELEX: Systematic evolution of ligands by exponential enrichment
SPE: Screen printed electrodes
MWCNTs: Multiwall carbon nanotubes
SAM: Self-assembled monolayers
GNI: Gross national income.
