# Supplementary material for: Potential Biomarkers and Their Applications for Rapid and Reliable Detection of Malaria
Source: Biomed Res Int. 2014 Apr 2;2014:852645. doi: 10.1155/2014/852645 (PMC3996934; doi:10.1155/2014/852645)
Supplement: Supplementary file 1 — Table S1 : List of PDB entries for Plasmodium lactate dehydrogenase. Table S2: List of inhibitors developed against lactate dehydrogenase from P.falciparum. [file 852645.f1.docx]

**SUPPLEMENTARY DATA**

**Title: Potential biomarkers and their applications for rapid and reliable detection of malaria**

Authors: Priyamvada Jain †, Babina Chakma †, Sanjukta Patra and Pranab Goswami*

Department of Biotechnology, Indian Institute of Technology Guwahati, Assam 781039, India

The supplementary material consists of list of PDB entries for Plasmodium lactate dehydrogenase, list of inhibitors developed against lactate dehydrogenase from *P.falciparum,*

Table S1 : List of PDB entries for Plasmodium lactate dehydrogenase.

| Serial no. | PDB ID | Name of entry^*^ | Resolution  (A°) | Reference |
| --- | --- | --- | --- | --- |
| 1 | 1CET | Chloroquine binds in the cofactor binding site of PfLDH | 2.05 | [37] |
| 2 | 1OC4 | LDH from *P.berghei* | 2.30 | [18] |
| 3 | 2AA3 | Crystal structure of PvLDH complex with APADH | 2.05 | [27] |
| 4 | 2A92 | Crystal structure of LDH from *P.vivax*: complex with NADH | 2.04 | [27] |
| 5 | 2A94 | Structure of PfLDH complexed to APADH | 1.50 | [27] |
| 6 | 1CEQ | Chloroquine binds in the cofactor binding site of PfLDH | 2.00 | [37] |
| 7 | 1U4O | PfLDH complexed with 2,6-naphthalenedicarboxylic acid | 1.70 | [35] |
| 8 | 1U4S | PfLDH complexed with 2,6-naphthalenedisulphonic acid | 2.00 | [35] |
| 9 | 1T2C | PfLDH complexed with NADH | 2.01 | [34] |
| 10 | 1LDG | PfLDH complexed with NADH and oxamateE | 1.74 | [17] |
| 11 | 1U5C | PfLDH complexed with 3,7-dihydroxynaphthalene-2-carboxylic acid and NAD+ | 2.65 | [35] |
| 12 | 1U5A | PfLDH complexed with 3,5-dihydroxy-2-naphthoic acid | 1.80 | [35] |
| 13 | 1T25 | PfLDH complexed with NADH and 3-hydroxyisoxazole-4-carboxylic acid | 1.90 | [34] |
| 14 | 1XIV | PfLDH complexed with 2-({4-chloro-[hydroxy(methoxy)methyl]cyclohexyl}amino)ethane-1,1,2-triol | 1.70 | [35] |
| 15 | 1T24 | PfLDH complexed with NAD+ and 4-hydroxy-1,2,5-oxadiazole-3-carboxylic acid | 1.70 | [34] |
| 16 | 1T2E | PfLDH S245A, A327P mutant complexed with NADH and oxamate | 1.85 | [34] |
| 17 | 1T2D | PfLDH complexed with NAD+ and oxalate | 1.10 | [34] |
| 18 | 1T26 | PfLDH complexed with NADH and 4-hydroxy-1,2,5-thiadiazole-3-carboxylic acid | 1.80 | [34] |

*Entries have been abbreviated for clarity of presentation

Table S2: List of inhibitors developed against lactate dehydrogenase from *P.falciparum*

| Serial no | Class of anti malarial | Most potent anti malarial found | Strain of *P.falciparum* | Mechanism of inhibition | IC_50_ value  (µM) | K_i_ (µM) | References |
| --- | --- | --- | --- | --- | --- | --- | --- |
| 1 | Gossypol and derivatives | Gossylic nitrile 1,1’-divalerate | FCB/NC1(chloroquine resistant)  CDC/I/HB3(chloroquine sensitive) | Competitive inhibition of NADH | 16  12 | 0.33  - | [12] |
| 2 | -do- | gossylic lactone(GL)  gossylic nitrile diacetate(GNDA)  Ability to selectively inhibit parasite LDH was studied. | in vitro anti plasmodium assay was not done | -do- | - | 1  1  GNDA is 100 times more selective | [23] |
| 3 | -do- | 7-p-trifluoromethylbenzyl-8-deoxyhemigossylic acid | -do- | -do- | - | 0.2 | [12] |
| 4 | -do- | 4-n-propyl dihydroxy napthoic acid | -do- | -do- | - | 0.3 | [33] |
| 5 | -do- | Gossylic lactone | -do- | -do- | - | 0.4 | [30] |
| 6 | Napthoic acid derivatives | 3,5-dihydroxy 2-napthoic acid | 3D7 |  | 1700 | - | [35] |
| 7 | Chloroquine | - | - | -do- | - | - | [36] |
| 8 | Chloroquine | - | - | -do- | - | - | [37] |
| 9 | 1,2,5-oxadiazole series(9 compounds)  1,2/1,5-isoxazole series(9 compounds)  1,2,5-thiadiazole series(3 compounds) | OXD1  IOA1  TDA1 | In vitro anti plasmodial activity was studied against 3D7(drug sensitive) and KI (drug resistant) strains for all three classes of compounds | Mixed inhibition against both NADH and pyruvate and competitive inhibition against lactate | 0.65^*^  1.1*  0.14* | 0.210  0.470  0.290 | [34] |
| 10 | A library of oxamic acid derivatives^$$^ | Compounds 5  Compound 6  Compound 17 | All derivatives were tested against W2(chloroquine resistant) and D6(mefloquine resistant) strains | Competitive inhibition of pyruvate | 9.41  8.40  43* | - | [3] |

*IC50 for pfLDH

$$ In this study no correlation was found between in vitro anti plasmodial assay and enzyme inhibition studies

.
